# Supplementary material for: Content-rich biological network constructed by mining PubMed abstracts
Source: BMC Bioinformatics. 2004 Oct 8;5:147. doi: 10.1186/1471-2105-5-147 (PMC528731; doi:10.1186/1471-2105-5-147)
Supplement: Additional File 2 — The original results of the above study (non-essential files are deleted to keep the file size under the limit set by BMC bioinformatics). [file 1471-2105-5-147-S2.bz2 › chilibotAdditionalFile2/dip10/707ID9482750E18044/html/MUTL_UVRD.html]

 


 **MUTL** and **UVRD** 
  
Found 29 abstracts in PubMed, retrieved 10.  
 

 What does Google say? 
 PDF only 
| .edu only 

---

**Interactive relationship** (e.g. stimulation, inhibition, etc)

**Neutral relationship**- The results indicate that  **MutL**  binds DNA and loads  **UvrD**  onto the DNA substrate.  Ref: 10984488 J Biol Chem, 2000
- Escherichia coli  **MutL**  loads DNA helicase II  [ **UVRD** ]  onto DNA.  Ref: 10984488 J Biol Chem, 2000
- Previous studies have shown that  **MutL**  physically interacts with  **UvrD**  DNA helicase II  [ **UVRD** ]  Hall, M.  Ref: 10984488 J Biol Chem, 2000
- A two hybrid screen previously identified a physical interaction between  **MutL**  and  **UvrD** .  Ref: 9880500 J Biol Chem, 1999
- All possible pairwise combinations of  **UvrD** ,  **MutL** , MutS, and MutH were tested using the yeast two hybrid system to identify potential interactions involving mismatch repair proteins.  Ref: 9880500 J Biol Chem, 1999
- As judged by protein affinity chromatography,  **MutL**  and  **MutL**  E32K both support formation of ternary complexes that also contain MutS and MutH or MutS and DNA helicase II  [ **UVRD** ] .  Ref: 10734142 J Biol Chem, 2000
- This phenotype is suppressed by a  **uvrD**  mutU defect, and partially suppressed by mutH,  **mutL** , or mutS mutations.  Ref: 11381137 Proc Natl Acad Sci U S A, 2001
- between  **MutL**  and  **UvrD**  are both important for stimulation of  **UvrD**  catalyzed unwinding.  Ref: 10984488 J Biol Chem, 2000

**Non-interactive relationship** (e.g. studied together, co-existance, homology, etc.)

- We also describe the alterations in the mutS,  **mutL**  and  **uvrD**  genes responsible for the mutator phenotype of hypermutable P. aeruginosa strains isolated from CF patients.  Ref: 11952911 Mol Microbiol, 2002
- Escherichia coli MutS,  **MutL** , and DNA helicase II  [ **UVRD** ]  are sufficient to initiate mismatch dependent unwinding of an incised heteroduplex Yamaguchi, M.  Ref: 9535911 J Biol Chem, 1998
- The increases in spontaneous reversion of histidine mutations were first measured in isogenic strains carrying mismatch repair defective mutH,  **mutL** , mutS, or  **uvrD**  alleles, relative to their mismatch repair proficient counterparts.  Ref: 9685594 Mutat Res, 1998
- The results shown here indicate that the putative P. aeruginosa mutS,  **mutL**  and  **uvrD**  genes are mutator genes.  Ref: 11952911 Mol Microbiol, 2002
- Seven out of the 11 mutator strains were found to be defective in the MMR system four mutS, two  **mutL**  and one  **uvrD** .  Ref: 11952911 Mol Microbiol, 2002
- Unwinding of both substrates in the presence of MutS,  **MutL** , DNA helicase II  [ **UVRD** ] , and single stranded DNA binding protein was mismatch dependent and initiated at the single strand break.  Ref: 9535911 J Biol Chem, 1998
- Here, we report the cloning and sequencing of two additional P. aeruginosa mismatch repair genes and the characterization, by complementation of deficient strains, of these two putative P. aeruginosa mismatch repair genes  **mutL**  and  **uvrD** .  Ref: 11952911 Mol Microbiol, 2002
- **MutL**  does not clamp  **UvrD**  onto the substrate.  Ref: 10984488 J Biol Chem, 2000
- The mismatch repair system mutS,  **mutL**  and  **uvrD**  genes in Pseudomonas aeruginosa molecular characterization of naturally occurring mutants.  Ref: 11952911 Mol Microbiol, 2002
- In Test A, repair proficient strain and its uvrA counterpart are used for detection of spontaneous and UV induced mutations, while in Test B mismatch repair deficient strains mutH, mutS,  **mutL**  and  **uvrD**  are used for amplified detection of spontaneous mutations caused by replication errors.  Ref: 9675243 Mutat Res, 1998
- The growth defect and filamentation phenotypes associated with 2 aminopurine exposure are effectively suppressed by null mutations in mutH,  **mutL** , mutS, or  **uvrD**  mutU, which encode activities that act upstream of the four exonucleases in the mechanism for the methyl directed reaction that has been proposed based on in vitro studies.  Ref: 11381137 Proc Natl Acad Sci U S A, 2001
- To investigate the effects on PV rates of mutations in genes for mismatch repair MMR, insertion deletion mutations of mutS,  **mutL** , mutH, dam, polI,  **uvrD** , mfd and recA were constructed in Hi strain Rd.  Ref: 11889052 EMBO J, 2002
